# Right Heart Remodeling in Patients with End-Stage Alcoholic Liver Cirrhosis: Speckle Tracking Point of View

**DOI:** 10.3390/jcm8091285

**Published:** 2019-08-22

**Authors:** Kun Zhang, Alexander Braun, Francisca von Koeckritz, Rosa B. Schmuck, Eva M. Teegen, Cesare Cuspidi, Frank Heinzel, Burkert Pieske, Marijana Tadic

**Affiliations:** 1 Department of Internal Medicine and Cardiology, Campus Virchow-Klinikum, Charité-Universitätsmedizin Berlin, Augustenburger Platz 1, 13353 Berlin, Germany; 2 Berlin Institute of Health (BIH), 13353 Berlin, Germany; 3 Department of Surgery, Campus Charité Mitte and Campus Virchow Klinikum, Charité-Universitätsmedizin Berlin, Augustenburger Platz 1, 13353 Berlin, Germany; 4 Clinical Research Unit, University of Milan-Bicocca and Istituto Auxologico Italiano IRCCS, Viale della Resistenza 23, 20036 Meda, Italy

**Keywords:** cirrhosis, right atrium, right ventricle, function, strain

## Abstract

Background: Data regarding cardiac remodeling in patients with alcoholic liver cirrhosis are scarce. We sought to investigate right atrial (RA) and right ventricular (RV) structure, function, and mechanics in patients with alcoholic liver cirrhosis. Methods: This retrospective cross-sectional investigation included 67 end-stage cirrhotic patients, who were referred for evaluation for liver transplantation and 36 healthy controls. All participants underwent echocardiographic examination including strain analysis, which was performed offline. Results: RV basal diameter and RV thickness were significantly higher in patients with cirrhosis. Conventional parameters of the RV systolic function were similar between the observed groups. Global, endocardial, and epicardial RV longitudinal strains were significantly lower in patients with cirrhosis. Active RA function was significantly higher in cirrhotic patients than in controls. The RA reservoir and conduit strains were significantly lower in cirrhotic patients, while there was no difference in the RA contractile strain. Early diastolic and systolic RA strain rates were significantly lower in cirrhotic patients than in controls, whereas there was no difference in the RA late diastolic strain rate between the two groups. Transaminases and bilirubin correlated negatively with RV global longitudinal strain and RV-free wall strain in patients with end-stage liver cirrhosis. The Model for End-stage Liver Disease (MELD) score, predictor of 3-month mortality, correlated with parameters of RV structure and systolic function, and RA active function in patients with end-stage liver cirrhosis. Conclusions: RA and RV remodeling is present in patients with end-stage liver cirrhosis even though RV systolic function is preserved. Liver enzymes, bilirubin, and the MELD score correlated with RV and RA remodeling.

## 1. Introduction

One decade ago, the term “cirrhotic cardiomyopathy” was first introduced, which usually refers to the left ventricular (LV) diastolic dysfunction, an inadequate LV response to the stress or prolonged QT interval, in the absence of known cardiac disease [1,2]. Most studies have been focused on LV functional remodeling and particularly LV diastolic dysfunction, which was usually determined by tissue Doppler [3,4]. In the meantime, new echocardiographic techniques that are able to detect subtle and subclinical changes in cardiac mechanics appeared. One of these new techniques is speckle-tracking imaging that enables determination of detailed myocardial mechanics during the whole cardiac cycle. Investigations showed important predictive value of both LV and right ventricular (RV) strains in patients with different pathologies [5,6,7].

There are only a few studies that investigated cardiac remodeling in cirrhotic patients, subjects with non-alcoholic fatty liver disease, or liver cirrhosis using strain analysis [4,8,9,10]. Data regarding an RV strain in the patients with liver cirrhosis are scarce [11]. There is no data regarding RV layer-specific changes and right atrial (RA) phasic function in the patients with end-stage liver cirrhosis. The majority of studies showed that RV function, assessed by conventional echocardiographic parameters, was preserved [4,8,9]. Nevertheless, the large number of patients with cirrhosis has symptoms of RV dysfunction. Therefore, we hypothesized that new echocardiographic parameters of RV dysfunction such as global and layer-specific strain could provide insight information regarding RV function and mechanics.

The aim of the present study was to evaluate RV structure, function, and mechanics, as well as RA phasic function, in patients who were in the terminal stage of liver cirrhosis. Additionally, we investigated the correlation between liver enzymes and Model for End-stage Liver Disease (MELD) score and echocardiographic parameters of RV and RA remodeling.

## 2. Methodology

This retrospective cross-sectional study included 103 participants: 67 patients with alcoholic liver cirrhosis preparing for liver transplantation and 36 control subjects of similar age and gender distribution. Cirrhosis patients were recruited from the department for liver transplantation between January 2012 and June 2015 and all patients underwent liver transplantation. Exclusion criteria were heart failure, coronary artery disease, atrial fibrillation, congenital heart disease, more than mild valvular heart disease, neoplastic disease, and renal failure. Controls were recruited from the echocardiography department at the same period among the patients who were referred to for a regular check-up examination, palpitations, or an innocent heart murmur. All subjects underwent echocardiographic examinations and analyses of obtained data were performed offline. The investigator who performed the analyses was blinded for groups. Subjects with inadequate echocardiographic images were excluded from the study (8 patients and 5 controls). The MELD score was calculated for cirrhotic patients that included creatinine, bilirubin, the International Normalized Ratio (INR), sodium, and information regarding hemodialysis (at least twice in the last week).

Anthropometric measures (height, weight) and laboratory analyses (liver enzymes, total bilirubin level, and creatinine level) were taken from all the study subjects. The body mass index (BMI) and body surface area (BSA) were calculated for each subject. BSA was calculated according to the formula: (height (cm) * weight (kg)/3600)^−1/2^. The study was approved by the local Ethics Committee.

### 2.1. Echocardiography

Echocardiographic examinations were performed using Vivid 7 ultrasound (GE Vingmed, Horten, Norway).

The RV internal diameter was determined in the basal RV segment in apical four-chamber view [12]. Linear measurement of RV-free wall thickness was performed in four-chamber subcostal view at 2DE zoomed image at end-diastole, below the tricuspid annulus at a distance approximating the length of the anterior tricuspid leaflet [12].

Tissue Doppler velocity during systole (s_t_) was evaluated in the apical four-chamber view [12]. Tricuspid annular plane systolic excursion (TAPSE) has been measured in all participants, according to the guidelines [12]. Fractional area change was calculated as the difference between RV end-diastolic and end-systolic area divided with the RV end-diastolic area [12]. RV systolic blood pressure (PASP) was measured in the patients with tricuspid regurgitation.

### 2.2. Right Ventricular Strain

2D strain imaging was performed by using three consecutive cardiac cycles in the apical four-chamber view that was focused on the RV [13]. EchoPAC 201 software (GE-Healthcare, Horten, Norway) was used for the 2D strain analysis.

The automatic tracking of the endocardial contour was performed in end-systole and it was carefully verified. The region of interest was manually corrected to ensure optimal tracking and inclusion of the entire RV thickness including endocardial, mid-myocardial, and epicardial layers. After delineating the region of interest, software allowed the investigation of three myocardial layers: endocardial, mid-myocardial, and epicardial. However, it is assumed that the mid-myocardial layer is equal to the global RV strain.

### 2.3. Right Atrium

RA volume was calculated by the single plane Simpson’s method of disks in apical four-chamber view. RA volumes (RAVs) were measured in three different sequences of the cardiac cycle. Maximal RAV was measured just before the tricuspid valve opening. In addition, pre-A RAV (pre-atrial contraction) was determined at the onset of atrial systole (peak of P wave in ECG), whereas minimal RAV was measured at the tricuspid valve closure. RA volumes were indexed for BSA and RAV indexes (RAVIs) were calculated for each study participant. The total emptying volume, which represents the RA reservoir function, was calculated as the difference between maximum and minimum RAV. Passive emptying volume, which represents the conduit function, was calculated as the difference between maximum and pre-A RAV, and active emptying volume, which corresponds to RA booster function, was calculated as the difference between pre-A and minimum RAV. Accordingly, total emptying fraction (EF) was calculated as the ratio between total emptying volume and maximum RAV. Passive EF was computed as the ratio between passive and maximum, and active EF was calculated as the proportion between active and pre-A RAV.

2D strain imaging was performed in the apical four-chamber apical view [14] and using commercially available software Echo PAC 201 (GE-Healthcare, Horten, Norway) was used for the 2D strain analysis. The RA strain was calculated using R-R gating. The RA endocardium was manually traced. Two peaks of RA strain corresponded to reservoir function (first peak between R wave and T wave) and atrial contractile function (starting on the P wave). The difference between the reservoir strain and atrial contractile strain values reflects the conduit function.

### 2.4. Statistical Analysis

Continuous variables were presented as mean ± standard deviation (SD), and the Student *t*-test was used to detect differences between the two groups when variables showed normal distribution, whereas the Mann-Whitney U test was used when they showed non-normal distribution. Differences in proportions were compared by using the χ². Spearman’s correlation coefficient was used for determining the correlation between different clinical and echocardiographic parameters in the patients with end-stage alcoholic liver cirrhosis. The inter-observer and intra-observer agreements were determined by evaluating the intra-class correlation coefficients (ICC) in 15 randomly chosen subjects. The *p* < 0.05 was considered statistically significant.

## 3. Results

Patients with cirrhosis were older than controls, whereas there was no difference in sex distribution and BMI between two groups (Table 1). As expected, all liver enzymes and bilirubin were significantly higher in cirrhosis patients than in controls.

### 3.1. Echocardiography

The RV basal diameter and RV thickness were significantly higher in patients with cirrhosis (Table 2). There was no difference in RV end-systolic and end-diastolic areas. Parameters of RV systolic function (TAPSE, FAC, s’) were similar between the observed groups (Table 2). Systolic pulmonary artery pressure (PAP) was significantly higher in patients with cirrhosis. Vena cava inferior diameter was similar between the observed groups.

Global, endocardial, and epicardial RV longitudinal strains were significantly lower in patients with cirrhosis (Table 2). The same results were found for the whole RV and RV-free wall.

### 3.2. RA Parameters

RA maximal area and volume index were significantly higher in cirrhosis patients (Table 3). Minimal and pre-A RAVIs were similar between controls and cirrhotic patients. Total and passive RA emptying fraction (RAEF) did not significantly differ between the observed groups, whereas active RAEF was significantly higher in cirrhotic patients than in controls (Table 3).

The RA reservoir and conduit strains were significantly lower in cirrhotic patients, while there was no difference in RA contractile strain (Table 3). Early diastolic and systolic RA strain rates were significantly lower in cirrhotic patients than in controls, whereas there was no difference in the RA late diastolic strain rate between two groups (Table 3).

### 3.3. Correlation Analysis

AST, ALT, AP, and bilirubin correlated negatively with the RV global longitudinal strain and the RV-free wall strain in patients with end-stage liver cirrhosis (Table 4). ALT correlated with the RA reservoir strain (Table 4). GGT correlated with PAP, the RA reservoir, and contractile strains. Bilirubin also correlated with the RA contractile strain (Table 4). The MELD score correlated with the RV basal diameter, TAPSE, s’, PAP, and RA active emptying fraction in patients with end-stage liver cirrhosis (Table 4).

### 3.4. Intra-Observer Variability

The intra-observer variability was high for RV global longitudinal strain: ICC = 0.91 (95%CI: 0.89–0.96, *p* < 0.001) as well as for RV global subendocardial longitudinal strain: ICC = 0.93 (95%CI: 0.87–0.97, *p* < 0.001). RV global subepicardial longitudinal strain: ICC = 0.84 (95%CI: 0.77–0.95, *p* < 0.001).

### 3.5. Inter-Observer Variability

The inter-observer variability was high for RV global longitudinal strain: ICC = 0.89 (95%CI: 0.84–0.95, *p* < 0.001). RV global subendocardial longitudinal strain: ICC = 0.90 (95%CI: 0.85–0.97, *p* < 0.001). RV global subepicardial longitudinal strain: ICC = 0.79 (95%CI: 0.71–0.89, *p* < 0.001).

## 4. Discussion

The present study revealed several important findings: (i) RV mechanics was impaired in patients with end-stage liver cirrhosis. (ii) All myocardial layers were affected in cirrhotic patients. (iii) RA phasic function was deteriorated in patients with cirrhosis when compared with controls. (iv) MELD score, as an important predictor of three-month mortality in patients with liver cirrhosis, correlated with parameters of RV remodeling and RA phasic function in cirrhotic patients.

RV global longitudinal strain, as well as endocardial and epicardial strains, was significantly lower in cirrhotic patients than in controls. The same results were obtained for the whole RV and RV-free wall. Reduced RV strain was also found in other investigations that included patients with cirrhosis or even patients with non-alcoholic fatty liver disease [11,15,16]. However, this is the first study that showed layer-specific changes in the RV strain in patients with end-stage liver cirrhosis. It must be emphasized that RV was larger and thicker in cirrhotic patients than in healthy controls, which potentially could contribute to the RV strain reduction in these patients. Rimbas et al. also found larger RV in cirrhotic patients than in controls, but the RV global longitudinal strain was still similar between patients and controls [11]. We did not find any difference in parameters of RV systolic function (TAPSE, FAC, s’) between patients and controls. Rimbas et al. agree with our results regarding RV systolic function in cirrhotic patients [11]. On the other hand, Sunbul et al. found significantly lower TAPSE and s’ in patients with non-alcoholic fatty liver disease [16].

Our findings indicate that all RV myocardial layers are equally affected in patients with cirrhosis. This represents a new finding that has not been previously investigated. The structure of the RV and number of layers in its wall is still a topic of debate. There is a lack of agreement if the RV wall consists of two or three layers. However, studies performed on the LV clearly showed that remodeling usually begins with endocardium, spreads on the mid-myocardium, and ends in the epicardial layer [17]. This pattern was first discovered in ischemic heart disease, but it seems that this pattern of myocardial impairment persists in other diseases such as arterial hypertension or diabetes [18,19]. Our study group showed that all RV myocardial layers were equally affected in patients with arterial hypertension and diabetes [20]. In the current study, we obtained similar results for cirrhotic patients.

The present study showed that the RA phasic function was deteriorated in the patients in end-stage alcohol cirrhosis than in healthy controls. Different results were obtained using volumetric and strain analysis of the RA phasic function. Namely, volumetric RA analysis showed no statistically important difference in the RA reservoir and conduit functions between patients and controls, whereas the RA contractile function was significantly higher in cirrhotic patients. Strain analysis showed significantly lower RA reservoir and conduit functions in cirrhotic patients and no difference in RA contractile function. Previous studies performed in the same or similar groups of patients did not assess the RA phasic function. It seems that strain analysis was more sensitive for detecting subtle changes in the RA phasic function in patients with cirrhosis. This is supported by the fact that more correlations were found between the strain-derived RA phasic function and liver enzymes than between the volumetric-derived RA phasic function and liver enzymes. This finding was important from a clinical point of view because volumetric RA phasic analysis is time consuming, whereas RA strain analysis is rapid analysis that provides all required parameters simultaneously. However, it must be emphasized that commercial software dedicated for RA strain analysis still does not exist and all available data were obtained using software dedicated for LV strain analysis.

RA dilatation and phasic function impairment have several important clinical implications. RA dilatation is associated with higher risk of atrial fibrillation recurrence than left atrial dilatation [21] as well as higher mortality risk in patients with pulmonary hypertension, pulmonary embolism, and heart failure [22]. Some authors showed that the RA strain represented a good noninvasive method for pulmonary hemodynamics assessment [23]. This could be important in patients with cirrhosis who had increased pulmonary pressure in the present study. However, one should be cautious in the interpretation of these results because not all investigations agree about the relationship between echocardiographic and hemodynamic evaluation of pulmonary pressure.

There are several possible mechanisms that could explain a reduced RV strain and impaired RA phasic function in cirrhotic patients. RV and RA strain changes are strongly associated with other parameters of right heart remodeling such as dilatation of the right heart, RV hypertrophy, and increased pulmonary pressure. However, it should be emphasized that neither of these structural, functional, or strain parameters reached pathological values, but they were deteriorated in comparison with healthy controls. Desensitization of β-adrenergic receptor, higher production of nitric oxide and carbon monoxide, increased concentration of inflammatory parameters such as interleukin-8, Interleukin-6, Interleukin-1b, TNF- α, and transforming growth factor- β are possible mechanisms that could explain impaired cardiac function in liver cirrhosis. Retention of salt and water, characteristic for cirrhosis, induces the activation of the renin-angiotensin-aldosterone system, which further provokes cardiac remodeling-cardiac hypertrophy, intramyocardial fibrosis, and fibrosis of intramyocardial arteries [24,25].

The most important clinical implication of this study is the necessity for more detailed echocardiographic assessment of the right heart in patients with liver cirrhosis. This particularly refers to the evaluation of RV and RA strain, which is currently not part of routine examination. The strain analysis is the rapid method that provides very useful information regarding RV and RA function and mechanics in a few minutes and it is important in situations when conventional echocardiographic parameters failed to show right heart impairment, which occurs often in cirrhotic patients, as demonstrated in our study. A recent study showed that increased RV afterload was related with increased hemodynamic complications and worse long-term survival after liver transplantation in the patients with end stage cirrhosis [26]. RV failure can lead to systemic hypoperfusion and splanchnic congestion, which both contribute to worse patient and graft outcomes. These are the reasons why the right heart function has a pivotal role in the prognosis of liver transplantation.

## 5. Limitations

Our study has several limitations. Sample size was limited and statistical significance was not reached in some important comparisons, even though we found a clear trend toward a significant difference. Moreover, this was a retrospective study and the echocardiographic exams were not optimized for research purposes, which resulted in exclusion of some subjects due to a lack of adequate echocardiographic images. All patients with cirrhosis underwent liver transplantation, but outcome was not known in all patients due to the hospital informative system that was changed three years ago, which resulted in a loss of a large amount of data. Therefore, the predictive value of the RV and RA strain was not possible to determine. Biomarkers related to RV or LV dysfunction were not available for all patients, which potentially could be helpful to define association between cirrhosis and cardiac remodeling. Lastly, alcohol by itself could induce cardiac dysfunction and we could not compare its influence with other etiologies because all our patients were diagnosed for alcoholic end-stage liver cirrhosis.

## 6. Conclusions

The RV structure and mechanics are impaired in patients with end-stage liver cirrhosis preparing for liver transplantation. The RV strain is reduced for the whole ventricle, but also for the RV-free wall, which indicates that cirrhosis has an independent effect on the RV. Conventional echocardiographic parameters of RV systolic function remained unchanged in patients with cirrhosis. RA phasic function is also impaired in patients with cirrhosis comparing with controls. Using volumetric and strain analysis of the RA phasic function, we obtained that all functions (reservoir, conduit, and contractile) are impaired in cirrhotic patients. Further follow-up studies with a larger number of patients are necessary to establish the predictive value of the RV strain and RA phasic function on the outcome in patients with end-stage liver cirrhosis.

## Figures and Tables

**Table 1:** Demographic and clinical parameters of the study population.

	Controls (*n* = 36)	Cirrhosis (*n* = 67)	*p*
Age (years)	47 ± 13	53 ± 12	0.039
Male (%)	16 (44)	33 (49)	0.683
BMI (kg/m^2^)	24.9 ± 3.8	26 ± 7.0	0.282
ALT (U/L)	25 ± 10	58 ± 45	<0.001
AST (U/L)	29 ± 15	80 ± 60	<0.001
AP (U/L)	66 ± 18	220 ± 180	0.001
GGT (U/L)	22 ± 12	128 ± 100	0.003
Creatinine (mg/dL)	0.80 ± 0.16	0.98 ± 0.55	0.056
Bilirubin (mg/dL)	0.5 ± 0.3	6.1 ± 8.4	<0.001
MELD	0.71 ± 1.2	13.5 ± 8.9	<0.001

ALT—alanine transaminase. AP—alkaline phosphatases. AST—aspartate transaminase. BMI—body mass index. GGT—gamma-glutamyl transferase. MELD—model for end-stage liver disease.

**Table 2:** Echocardiographic parameters of the study population.

	Controls (*n* = 36)	Cirrhosis (*n* = 67)	*p*
Right ventricle			
RV basal diameter (mm)	36 ± 6.8	39 ± 7.3	0.029
RV thickness (mm)	4.4 ± 0.8	5.0 ± 1.0	0.023
RV EDA (cm^2^)	17.6 ± 4.3	19.0 ± 5.2	0.171
RV ESA (cm^2^)	9.4 ± 2.7	9.7 ± 2.5	0.596
FAC (%)	47 ± 8	48 ± 10	0.483
TAPSE (mm)	25 ± 4	25 ± 5	0.809
s_t_ (cm/s)	13 ± 2.6	14 ± 2.8	0.597
PAPs (mmHg)	22 ± 5	27 ± 8	0.025
IVC (mm)	15 ± 3	17 ± 4	0.160
Right ventricular strain (%)			
RV global (%)	21.8 ± 1.7	19.8 ± 4.2	0.005
RV free wall (%)	26.5 ± 3.1	23.4 ± 5.9	0.004
RV longitudinal multilayer strain for the whole ventricle (%)			
Endocardial	25.0 ± 2.1	23.0 ± 4.8	0.020
Epicardial	19.3 ± 2.0	17.4 ± 4.3	0.010

EDA—end-diastolic area. ESA—end-systolic area. FAC—fractional area change. IVC—vena cava inferior. PAPs—systolic pulmonary pressure. RV—right ventricle. s’—systolic flow velocity across the lateral segment of tricuspid annulus. TAPSE-tricuspid annular plane systolic excursion.

**Table 3:** Right atrial parameters in the study population.

	Controls (*n* = 36)	Cirrhosis (*n* = 67)	*p*
RA volume analysis			
RAA (cm^2^)	13 ± 3.3	16 ± 4.8	0.001
RAVI_max_ (mL/m^2^)	17.2 ± 6.6	23.0 ± 10.2	0.005
RAVI_min_ (mL/m^2^)	8.3 ± 3.9	10.0 ± 6.1	0.093
RAVI_pre-a_ (mL/m^2^)	11.2 ± 5.2	13.9 ± 7.2	0.076
RA TotEF (%)	52 ± 12	57 ± 14	0.063
RA PassEF (%)	35 ± 18	35 ± 18	0.882
RA ActEF (%)	26 ± 13	33 ± 16	0.022
RA strain analysis			
RA reservoir strain (%)	46 ± 10	39 ± 18	0.020
RA conduit strain (%)	27 ± 8	20 ± 10	<0.001
RA contractile strain (%)	19 ± 7	19 ± 11	0.964
RA early diastolic strain rate (1/s)	−1.5 ± 0.7	−1.1 ± 0.7	0.003
RA late diastolic strain rate (1/s)	−2.4 ± 1.1	−2.1 ± 1.1	0.155
RA systolic strain rate (1/s)	2.1 ± 0.5	1.8 ± 0.8	0.045

BSA—body surface area. EF—emptying fraction. RA—right atrium. RAVI—right atrial volume index.

**Table 4:** Correlation analysis in the patients with cirrhosis.

	AST (U/L)	ALT (U/L)	AP (U/L)	GGT (U/L)	Bilirubin (mg/dL)	MELD
	r	r	r	r	r	r
RV basal diameter (mm)	0.101	−0.008	−0.014	0.100	0.143	0.268 *
RV thickness (mm)	0.007	0.186	−0.037	−0.142	0.077	0.079
TAPSE (mm)	0.228	0.116	−0.145	−0.212	0.246	0.357 *
s_t_ (cm/s)	0.089	0.048	−0.068	−0.234	0.106	0.375 *
FAC (%)	0.106	0.087	0.162	−0.075	0.214	0.183
PAPs (mmHg)	0.016	0.077	−0.032	−0.325 *	0.237	0.345 *
RV global longitudinal strain (%)	−0.310 *	−0.303 *	−0.374 *	0.016	−0.371 *	−0.228
RV free wall longitudinal strain (%)	−0.333 *	−0.325 *	−0.353 *	0.041	−0.360 *	−0.202
RA TotEF (%)	0.026	0.083	0.044	−0.065	−0.039	−0.076
RA PassEF (%)	0.060	−0.049	0.077	0.084	0.032	0.093
RA ActEF (%)	−0.026	0.122	0.028	−0.135	−0.088	−0.261 *
RA reservoir strain (%)	0.114	−0.243 *	−0.055	−0.273 *	0.179	0.047
RA conduit strain (%)	0.076	0.196	−0.043	−0.178	0.098	0.036
RA contractile strain (%)	0.089	0.188	−0.011	−0.285 *	0.174 *	0.018

*—*p* < 0.05, **—*p* < 0.01. ALT—alanine transaminase. AP—alkaline phosphatases. AST—aspartate transaminase. BMI—body mass index. EF—emptying fraction. FAC—fractional area change. GGT—gamma-glutamyl transferase. MELD—model for end-stage liver disease. IVC—vena cava inferior. PAPs—systolic pulmonary pressure. RA—right atrium. RV—right ventricle. s’—systolic flow velocity across the lateral segment of tricuspid annulus. TAPSE—tricuspid annular plane systolic excursion.
